# Early Choriocapillaris Loss in a Porcine Model of RPE Cell Debridement Precedes Pathology That Simulates Advanced Macular Degeneration

**DOI:** 10.1167/iovs.65.4.8

**Published:** 2024-04-03

**Authors:** Raymond Iezzi, Brittni A. Scruggs, Jarel Gandhi, Francesca N. Zenti, Noah Shafi, Aubrey Berger, Alan D. Marmorstein

**Affiliations:** 1 Department of Ophthalmology, Mayo Clinic, Rochester, Minnesota, United States; 2 Regenerative Sciences, Mayo Clinic Graduate School of Biomedical Sciences, Rochester, Minnesota, United States

**Keywords:** advanced macular degeneration, choroidal neovascularization, geographic atrophy, porcine model, retinal pigment epithelium

## Abstract

**Purpose:**

No large-mammal surgical models exist for geographic atrophy (GA), choroidal neovascularization (CNV), and pachychoroidal vascular remodeling. Our goal was to develop a porcine RPE debridement model of advanced macular degeneration to study photoreceptor cell loss and choroidal remodeling.

**Methods:**

Seven 2-month-old female domestic pigs were used for this study. After 25G vitrectomy, the area centralis was detached via subretinal bleb. A nitinol wire (Finesse Flex Loop) was used to debride RPE cells across a 3- to 5-mm diameter region. Fluid–air exchange was performed, and 20% SF_6_ gas injected. Animals underwent fundus photography, fluorescein angiography, optical coherence tomography (OCT), and OCT-angiography (OCTA) at 2 weeks, 1 month, 2 months, 3 months, and 6 months postoperatively. Retinal histology was obtained at euthanasia, 2 months (*n* = 3), 3 months (*n* = 2), or 6 months (*n* = 2) after surgery.

**Results:**

RPE debridement resulted in GA with rapid loss of choriocapillaris, progressive loss of photoreceptors, and pachychoroidal changes in Sattler's and Haller's layers in all seven eyes undergoing debridement within 2 months. OCT and histological findings included subretinal disciform scar with overlying outer retinal atrophy; outer retinal tubulations and subretinal hyper-reflective material. OCTA revealed type 2 CNV (*n* = 4) at the edges of the debridement zone by 2 months, but there was no significant exudation noted at any time point.

**Conclusions:**

Surgical debridement of the RPE results in GA, CNV, and pachychoroid and reproduced all forms of advanced macular degeneration. This surgical model may be useful in examining the role of RPE and other cell replacement in treating advanced macular disease.

AMD is the leading cause of irreversible vision loss among the elderly population worldwide.^1^^,^^2^ Geographic atrophy (GA), the advanced form of nonexudative (dry) AMD, is characterized by the progressive degeneration of the RPE and the underlying choriocapillaris, leading to severe visual impairment owing to central scotoma.^3^^,^^4^ Similarly, inherited macular dystrophies, such as Stargardt disease, central areolar choroidal dystrophy, and mitochondrial disorders (e.g., maternally inherited diabetes and deafness), among others, lead to profound central vision loss due to progressive macular degeneration in advanced stages. Stressors, genetic variants, chronic inflammation, and complement pathways have been implicated in macular degeneration pathobiology,^5^^–^^7^ yet the temporal relationship of RPE loss, photoreceptor (PR) degeneration, and choroidal thinning remains elusive.

One approach to the study of GA has been the use of RPE debridement models. Although rodent,^8^^–^^11^ rabbit,^12^^–^^14^ and feline^15^ models have been instrumental in elucidating certain aspects of macular degeneration pathogenesis, their anatomical and physiological differences from humans limit their translational relevance. Thus, many investigators have turned to either pigs or nonhuman primates. Across all mammalian eyes, the pig eye resembles the human eye second only to primates, both of which have holangiotic retinal vasculature.^16^^,^^17^ Although a pig does not have a true macula, it does have a cone-rich central region of the retina that functions similarly to a human macula, allowing good daylight visual acuity and excellent color vision. Thus, the pig, like nonhuman primates, is a suitable model for macular and retinal degeneration research.

The first porcine RPE debridement model was introduced in the mid 1990’s by Del Priore et al.^18^^,^^19^ This model used subretinal mitomycin C and edetic acid to debride the RPE, resulting in choriocapillaris loss after RPE loss. The model was hampered by RPE repopulation, which limited its use in further studies. Sodium iodate has been used to trigger a chemically induced RPE and PR degeneration in many species including swine, but does not target the RPE selectively.^20^ Mechanical RPE debridement permits more selective targeting of the RPE and can be performed regionally in a large animal eye. Seah et al.^21^ performed mechanical RPE debridement in a nonhuman primate model to examine the efficacy of an RPE cell transplantation; however, they did not study the effects of RPE removal on the adjacent outer retina and choroid. Similarly, in another study using nonhuman primates, confluent macular laser was shown to cause retina and choroidal damage; however, laser-induced damage makes it difficult to ascertain timing of RPE, PR, and choroidal changes.^22^ Sharma et al.^23^ used a similar laser model in swine to examine the efficacy of RPE transplantation, but like Seah et al.,^21^ they did not study the effects of RPE removal on the adjacent outer retina and choroid.

This study presents the development of a porcine surgical model mimicking advanced macular degeneration using a mechanical RPE debridement technique. The retinal vascular structure of pigs closely mimic humans, making them an ideal choice for studying retinal diseases and regenerative therapeutic approaches.^18^^,^^20^^,^^24^ RPE debridement allows investigation of the pathophysiological changes associated with macular atrophy, including the extent and timing of choroid and PR loss after selective RPE loss. A large animal RPE debridement model may be useful in examining the role of RPE and other cell replacement in treating advanced macular disease. This study also investigates the timing and pathophysiology of macular neovascular complexes (MNCs) in the setting of GA. MNCs, characterized by abnormal blood vessel growth in the macula, are a significant cause of vision loss in patients with AMD. However, the specific mechanisms and temporal relationship between MNCs and GA development remain poorly understood.

Last, the impact of RPE loss on choroidal flow and its subsequent effects on vessel density and thickness remain unknown. Understanding these dynamic changes in the choroid is crucial, because the choroid plays a vital role in maintaining retinal homeostasis and is implicated in the progression of GA. By using a debridement pig model with selective and permanent RPE removal, we can assess these parameters clinically using OCT and OCTA, providing further insights into the pathophysiology of GA and pachychoroid while complementing existing animal models.

## Methods

### Animals

All animal procedures were approved by the Institutional Animal Care and Use Committee of Mayo Clinic and conducted in accordance with the ARVO Statement for the Use of Animals in Ophthalmic and Vision Research. Seven 2- to 3-month-old female domestic pigs (*Sus scrofa domesticus*), at an approximate weight of 28 to 32 kg that were purpose bred for medical research were used for this study.

### Vitrectomy and RPE Debridement

Surgeries were performed essentially as before^25^ with the following modifications. Before pars plana vitrectomy, indirect laser photocoagulation was performed 360° in the retinal periphery, anterior to the equator using a head-mounted 532 nM wavelength indirect laser ophthalmoscope (Purepoint, Alcon, Fort Worth, TX) to minimize the risk of post–pars plana vitrectomy retinal detachment. A 1- to 5-mm lateral canthotomy was performed on the right eye to improve exposure, and a 5-mL retrobulbar block of cefazolin (100 mg/mL) was administered into the subtenon's space superotemporally to proptose and stabilize the eye.

Three-port triamcinolone-assisted 25G pars plana vitrectomy using a Constellation Vitrectomy System (Alcon) and wide-angled, noncontact Biom fundus lens (Oculus Surgical, Wetzlar, Germany) was performed by one of two vitreoretinal surgeons (RI or BAS) with careful separation of the posterior hyaloid in the area centralis. Eye pressure was maintained using a balanced salt solution (BSS) infusion. A 38G polyimide subretinal cannula (38g PolyTip, MedOne, Sarasota, FL) was used to create a subretinal bleb using BSS with eye pressure decreased to 10 mm Hg. Injection of BSS was performed per surgeon preference, either with slow manual injection using a skilled assistant or with pneumatic foot-pedal control using the viscous fluid injector setting on the Constellation, with care to not exceed 12 pounds per square inch.

Endodiathermy was applied along the proximal bleb, and a 2-mm retinotomy was created with vertical scissors. A Finesse Flex Loop (Alcon) was used to gently debride and remove a 3- to 5-mm diameter region of RPE cells. Fluid–air exchange was performed, and 20% SF_6_ gas was injected. The sclerotomies were sutured with 8-0 Vicryl sutures, when indicated, to ensure airtight closure of all sclerotomy sites. The canthus was sutured with 4-0 chromic gut interrupted sutured. Gentamicin 0.3% drops were topically administered to the right eye three times daily for 5 days postoperatively.

### Color Fundus Photography, Fluorescein Angiography, Optical Coherence Tomography (OCT), and OCT Angiography (OCTA)

The pig was anesthetized using isoflurane as detailed in Gandhi et al.,^25^ for each postoperative examination. Eye drops were instilled for dilation and topical anesthesia as detailed elsewhere in this article. Color fundus photos, OCT, and OCTA were performed at 2 weeks, 1 month, 2 months, 3 months, and 6 months postoperatively as indicated in Table. For six pigs, the total area of pixels signaling nonzero flow within a predefined 0.5-mm diameter (0.2 mm^2^) measurement circle was recorded using Optovue flow software within the debridement zone and immediately adjacent to the debridement zone 2 months after surgery.

**Table.** PostOperative Clinical, OCT, and OCTA Findings for Pigs in This Study

Pig Number	22P974	22P009	22P230	21P424	21P425	21P427	21P428
Duration (months)	2	2	2	6	3	3	6
Disciform scar	+	+	+	+	−	−	+
GA	+	+	+	+	+	+	+
CNV type 1	−	−	−	−	−	−	−
CNV type 2	+	+	+	+	−	−	−
cRORA	+	+	+	+	+	+	+
iRORA	−	+	−	−	−	−	−
ORT	+	+	+	+	−	−	−
Pachyvessels	+	+	+	+	+	+	+
Thinning of inner choroid	+	+	+	+	+	+	+
Complete choroidal atrophy	−	+	−	+	−	+	+
ORAOS	+	+	+	+	−	−	−
Inner retinal atrophy	−	−	−	−	+	−	+
SRHRM	+	+	+	+	+	+	+
Pigment migration	−	+	+	+	+	+	+
Hypertransmission	+	+	+	+	+	+	−

cRORA, complete loss of RPE and outer retinal layers; iRORA, incomplete loss of RPE and outer retinal layers; ORAOS, outer retinal atrophy overlying scar; ORT, outer retinal tubulation; SRHRM, subretinal hyper-reflective material.

Fundus photographs were obtained using a custom-made video indirect ophthalmoscope. Images were processed using Photoshop (Adobe, San Jose, CA). Fluorescein angiography was performed by administering 2.5 mL of fluorescein (10%) intravenously and images were obtained using a custom-made video indirect ophthalmoscope, fitted with excitation and barrier filters suitable for fluorescein angiography. Images were acquired ≤10 minutes after dye administration. OCT and OCTA were performed using the Optovue Avanti OCT Angiovue System (Visionix, North Lombard, IL).

### Histology

Pigs were euthanized by rapid intravenous injection of a pentobarbital solution *FATAL-PLUS* (Vortech; 1 mL/10 lbs body weight) at 2 months (*n* = 3), 3 months (*n* = 2), or 6 months (*n* = 2) postoperatively (Table). Eyes were enucleated and immersed in Davidson’s cold fixative for >4 hours and then stored in neutral buffered formalin or PBS for later processing. Eyes were embedded in paraffin and sectioned at 10 µm as described previously.^25^ Sections were stained with hematoxylin and eosin (H&E) and photographed using a Nikon E-600 microscope with a color CCD camera and NIS-Elements Software (Nikon, Tokyo, Japan).

For immunohistochemistry, pig eyes were fixed by immersion in Davidson's fixative for 24 hours then transferred to neutral buffered formalin for 72 hours before processing into paraffin. Immunohistochemical staining was performed on 5-µm sections, as described previously,^26^^,^^27^ using the following antibodies: Rabbit anti-IAB1 (Invitrogen, Carlsbad, CA), Rabbit anti-Collagen IV (Novus Biologicals, Centennial, CO), or Rabbit anti-Glial Fibrillary Acidic Protein (Novus Biologicals). Antibodies were detected using a Vectastain Elite ABC kit with Vector VIP substrate (Vector Laboratories, Burlingame, CA), which generates a purple product. Nuclei were counterstained with methyl green (Vector Laboratories).

## Results

### Intraoperative Procedures

**Figure 1.**
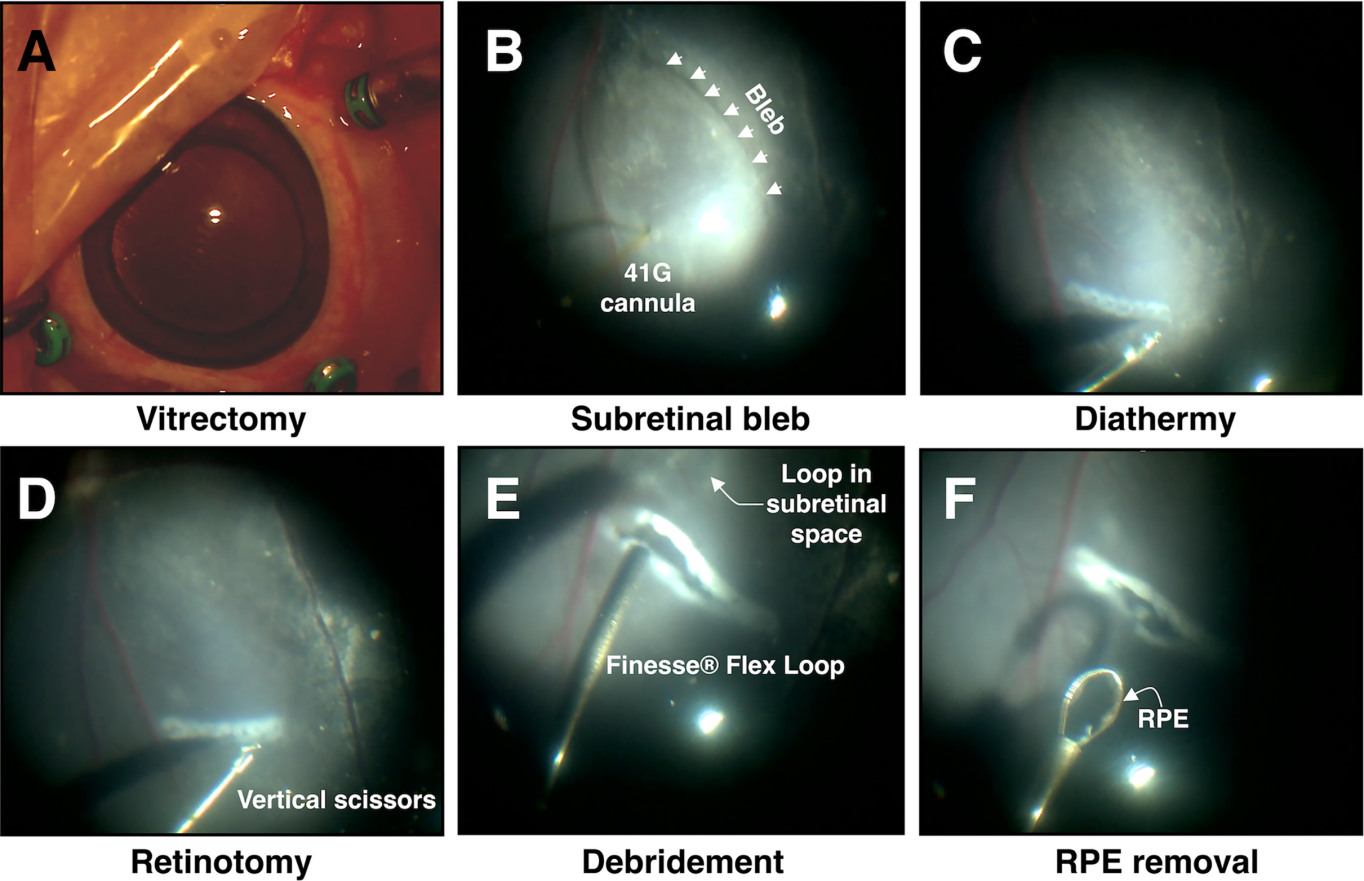
Intraoperative images documenting RPE debridement surgical steps in the porcine model. (**A**) Step 1: The 25G valved cannulas are placed 2.75 mm posterior to the limbus superonasally, inferonasally, and superotemporally for three-port lens-sparing vitrectomy of the right eye. Infusion line is seen superotemporally. The pig is positioned on left side with snout directed at surgeon. (**B**) Step 2: A posterior subretinal bleb (*white arrows*) is formed with BSS using a 38G cannula. (**C**) Step 3: Endodiathermy is used to apply cautery to the retinal surface between major arcade vessels in region of the bleb. (**D**) Step 4: Vertical scissors are used to create a linear retinotomy. (**E**) Step 5: A Finesse Flex Loop with a nitinol edge is inserted into the subretinal space to carefully remove RPE cells from the eye. (**F**) Step 6: RPE cells are removed until a large central debridement zone is visualized. Case concludes with an air–fluid exchange, light laser at the border of the retinotomy, 20% SF_6_ gas fill, and sclerotomy closure. BSS, balanced salt solution.

Vitreoretinal surgery on the pig requires specific modifications (with respect to human surgery) to avoid iatrogenic retinal detachments, lens touch, and postoperative complications.^28^ Our team has published previously on challenges in adapting to a pig model of subretinal surgery.^25^^,^^28^ Figure 1 shows the main surgical steps of the RPE debridement protocol (Supplementary Video S1). A three-port core vitrectomy with triamcinolone-assisted separation of the posterior hyaloid was performed with partial peripheral shave (Fig. 1A). A subretinal bleb was then performed posteriorly with BSS (Fig. 1B) before retinotomy creation (Figs. 1C, 1D) and careful removal of the RPE in the cone-rich region (Figs. 1E, 1F). The debridement zone measured ≥2-disc diameters (approximately 3–5 mm) for each pig. In contrast with primates, the pig does not have a pars plana, so there is a greater risk of intraoperative hemorrhage and/or retinal perforation solely with trocar placement. We intentionally did not perform a peripheral vitrectomy on any pig so as to provide vitreous support to the peripheral retina. The trocars must be placed according to age and weight of the pig and at an angle that avoids touching the very large crystalline lens. A retrobulbar or subtenon's block is needed before placing trocars to stabilize the eye, which has a propensity to be ballotable. A lateral canthotomy is imperative to increase exposure.

### PostOperative Course and Phenotypes

**Figure 2.**
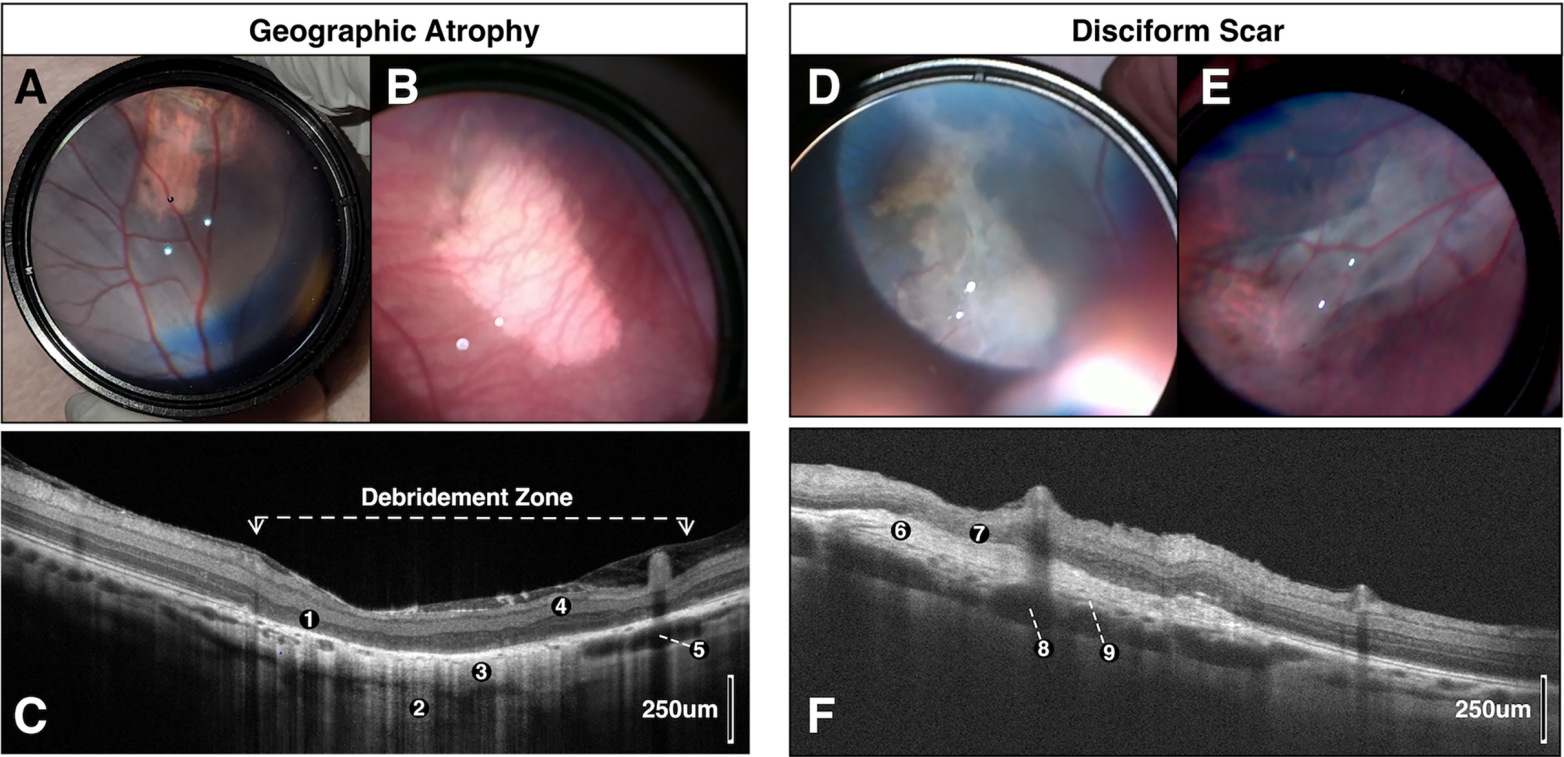
Two main clinical phenotypes identified two months after RPE debridement in the porcine model. (**A** and **B**) Indirect ophthalmoscopic images show large areas of central GA and pigmentary changes adjacent to the prior retinotomy sites of two different pigs. Prominent choroidal vasculature is seen in areas of debridement. (**C**) B-scan cross-sectional OCT image through the debridement zone (*dotted line*) two months after surgical debridement of the pig in (**A**). OCT findings included (1) cRORA, (2) hypertransmission defects, (3) loss of choroidal vasculature, (4) preserved inner retinal layers, and (5) several pachyvessel near edge of GA in Haller's layer. (**D**, **E**) Indirect ophthalmoscopic images of two different pigs show large, elevated disciform scars surrounded by pigmentary changes and GA. (**F**) B-scan cross-sectional OCT image through the debridement zone 2 months after surgical debridement of the pig in (**E**). OCT findings included (6) subretinal disciform scar with (7) overlying outer retinal atrophy, (8) numerous pachyvessels in Haller's layer beneath area of scar, and (9) thinning of inner choroidal vasculature. cRORA, complete RPE and outer retina atrophy.

Color fundus photographs, OCT, and OCTA were performed at 2 weeks, 1 month, 2 months, 3 months, and 6 months postoperatively (Table). By week 2, the gas bubble had dissipated in all pigs. The retina appeared flat, and there were no intraretinal or subretinal hemorrhages, subretinal fluid, cataract, inflammation, or retinal detachment noted. Postoperative clinical, structural OCT, and OCTA findings are summarized in Table for each pig. We observed two clinical phenotypes that were identified as early as the 2-week postoperative examination: (1) GA with complete loss of RPE but without scar formation (*n* = 2; Figs. 2A–C) and (2) disciform scar formation (*n* = 5; Figs. 2D, 2E, 6G).

### Evaluation of RPE Debridement Zone Using OCT and OCTA

**Figure 3.**
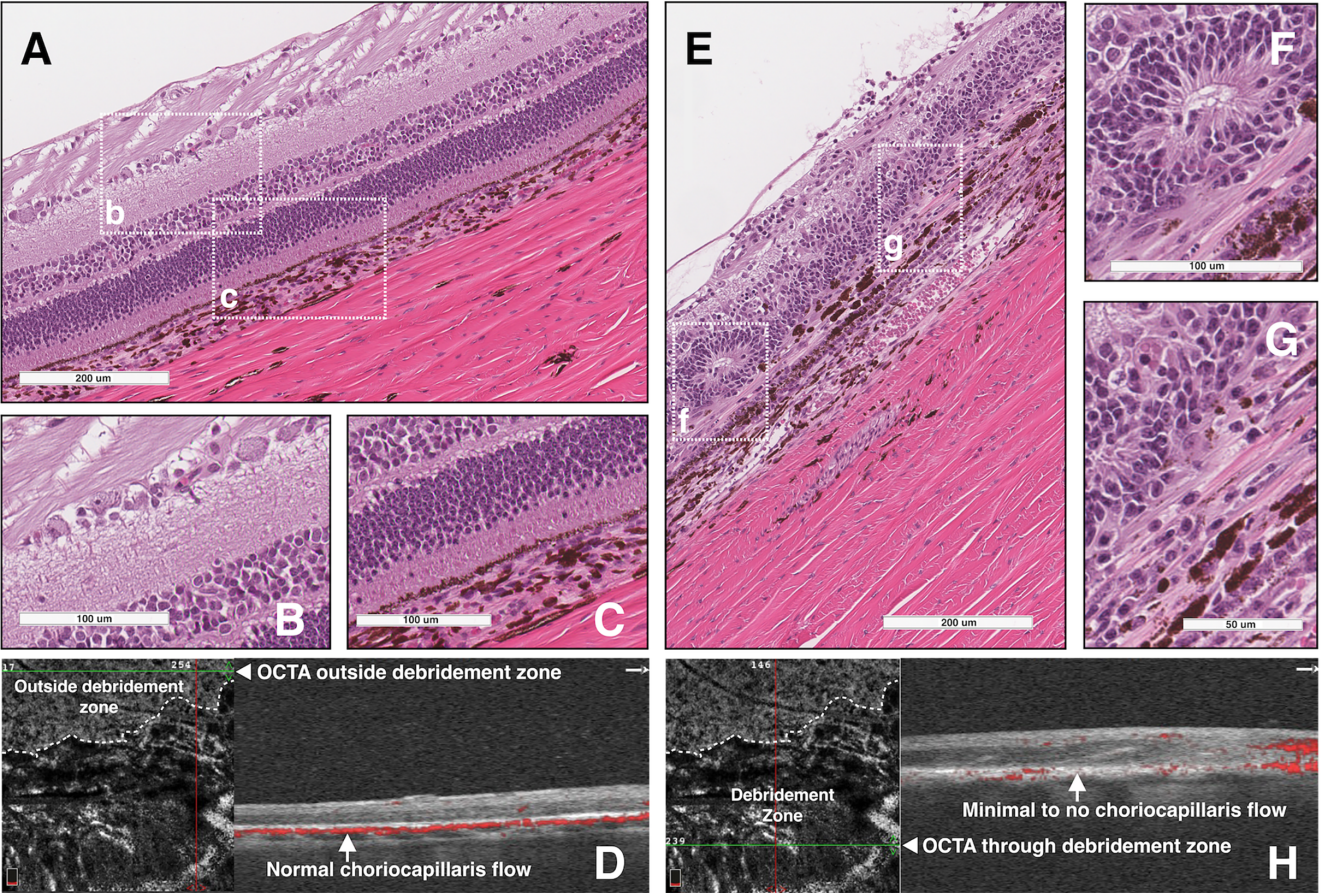
Disciform scar formation, outer retinal tubulation, PR loss, and decreased choriocapillaris flow in debridement zone. (**A**) H&E staining shows normal retinal anatomy outside the debridement zone. Higher magnification images of the inner retina (**B**) and outer retina and RPE (**C**) are shown; regions in the insets are demarcated by white dotted rectangles in (**A**). (**D**) OCTA choriocapillaris flow showed no abnormalities in the nondebrided region (*green line*). Corresponding structural OCT and OCTA flow overlay demonstrate normal flow signal throughout the choriocapillaris (inner choroid, *arrow*) through the area of nondebrided retina. (**E**) H&E staining shows significant anatomic changes throughout debridement zone, consistent with GA. Higher magnification images show an outer retinal tubulation (**F**) and evidence of RPE and PR loss, outer retinal disorganization with INL and ONL collapse, subretinal deposits, and pigment migration (**G**). Choriocapillaris loss is evident in the areas of debridement (**E**–**G**). Regions in the insets are demarcated by white dotted rectangles in (**E**). (**H**) Corresponding structural OCT and OCTA flow overlay show significant choriocapillaris flow loss (*arrow*) throughout debridement zone 2 months after surgery in the area of GA (*green line*). INL, inner nuclear layer; ONL, outer nuclear layer.

**Figure 4.**
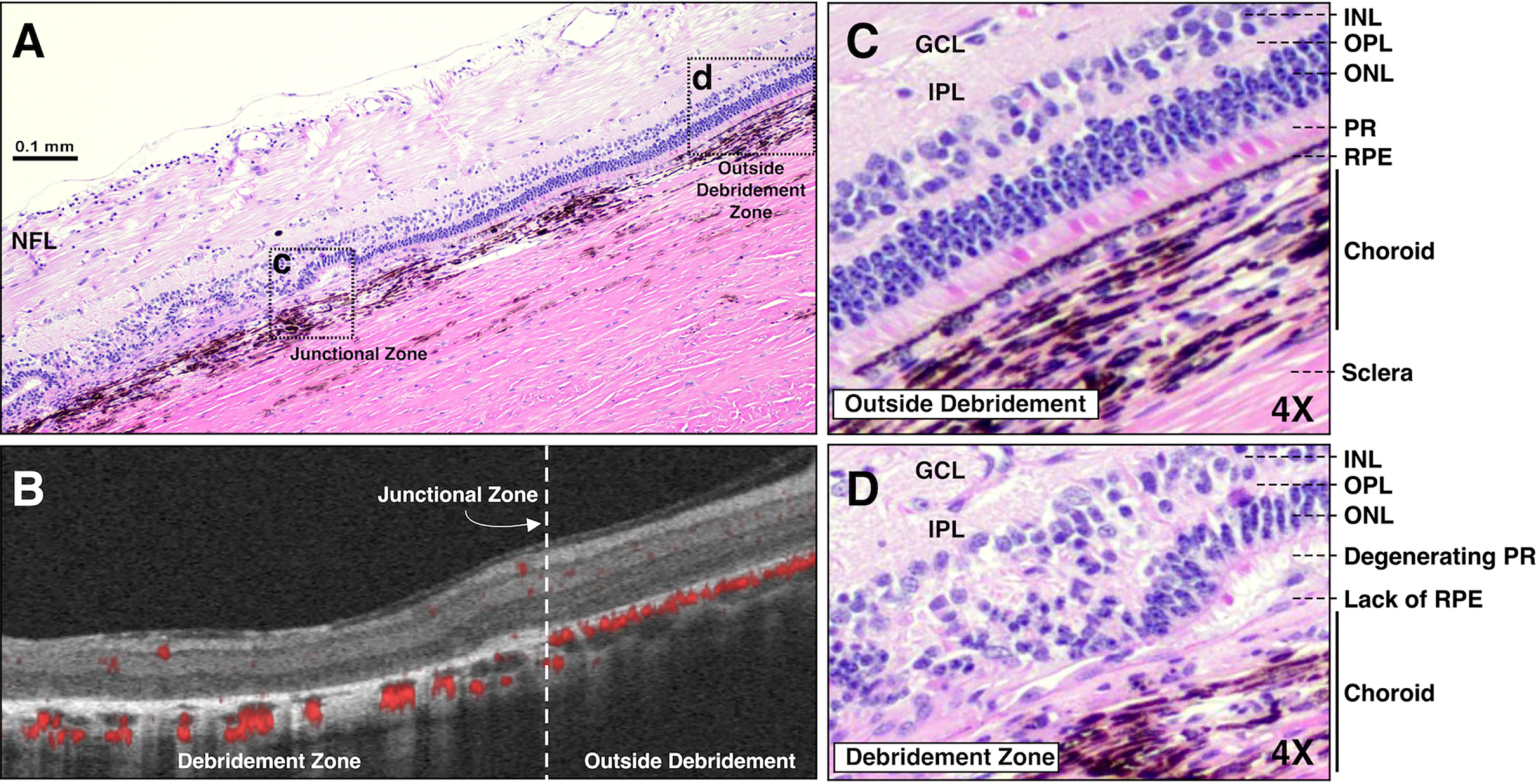
Outer retina degeneration and choroidal thinning in areas of RPE debridement are consistent with GA pathology. (**A**) H&E staining shows normal retinal anatomy outside the debridement zone (right of junctional zone) and significant outer retina and choroidal disruption within the debridement zone (left of junctional zone) 2 months after surgery. (**B**) Structural OCT and OCTA flow overlay show severe choriocapillaris flow loss underlying the debridement zone and normal choriocapillaris flow in the adjacent nondebrided region. (**C**, **D**) Higher magnification H&E images of the outer retina through nondebrided retina (**C**) and debridement zone (**D**) are shown; regions in the insets are demarcated by black dotted rectangles in (**A**). Note the degenerating PRs and collapsing INL and ONL layers in the atrophic region (**D**). GCL, ganglion cell layer; INL, inner nuclear layer; IPL, inner plexiform layer; NFL, nerve fiber layer; ONL, outer nuclear layer; OPL, outer plexiform layer.

**Figure 5.**
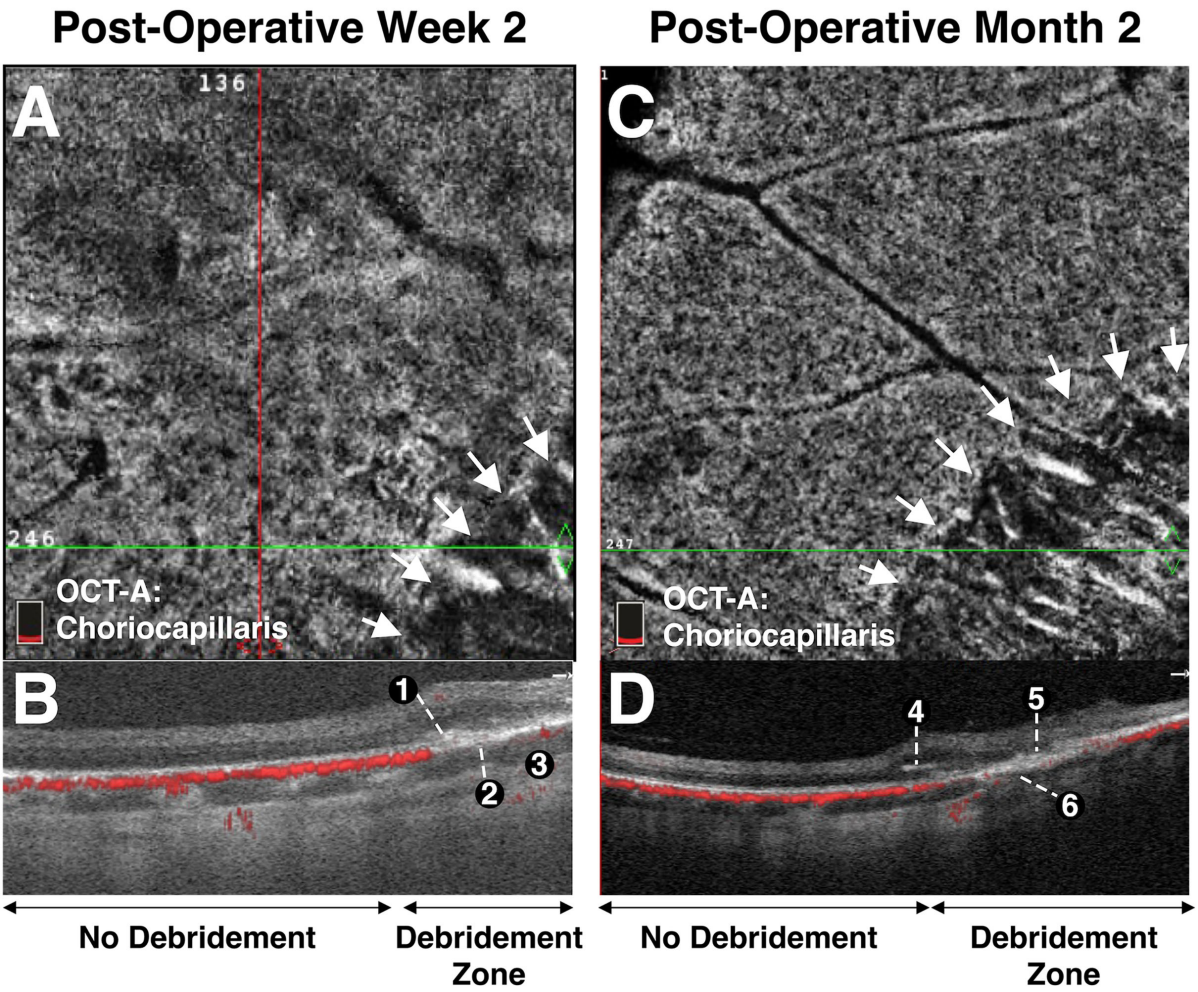
OCTA identifies early choriocapillaris loss and progressive outer retinal atrophy after RPE debridement. (A) OCTA identifies a choriocapillaris flow deficit in the area of debridement (white arrows) as early as two weeks after surgery, consistent with GA pathology. (**B**) Corresponding structural OCT and OCTA flow overlay show subretinal hyper-reflective material at junctional site (1), significant choriocapillaris flow loss throughout debridement zone (2) with preserved larger choroidal vessels in this region (3). (**C**) OCTA choriocapillaris flow deficit noted in area of debridement (*white arrows*) 2 months after surgery. (**D**) Corresponding structural OCT and OCTA flow overlay show subretinal hyper-reflective material with interval pigment migration at junctional site (4), interval worsening with complete atrophy of outer retinal layers in debridement zone (5), and significant choriocapillaris flow loss throughout the debridement zone with interval loss of larger choroidal vessels in this region (6).

**Figure 6.**
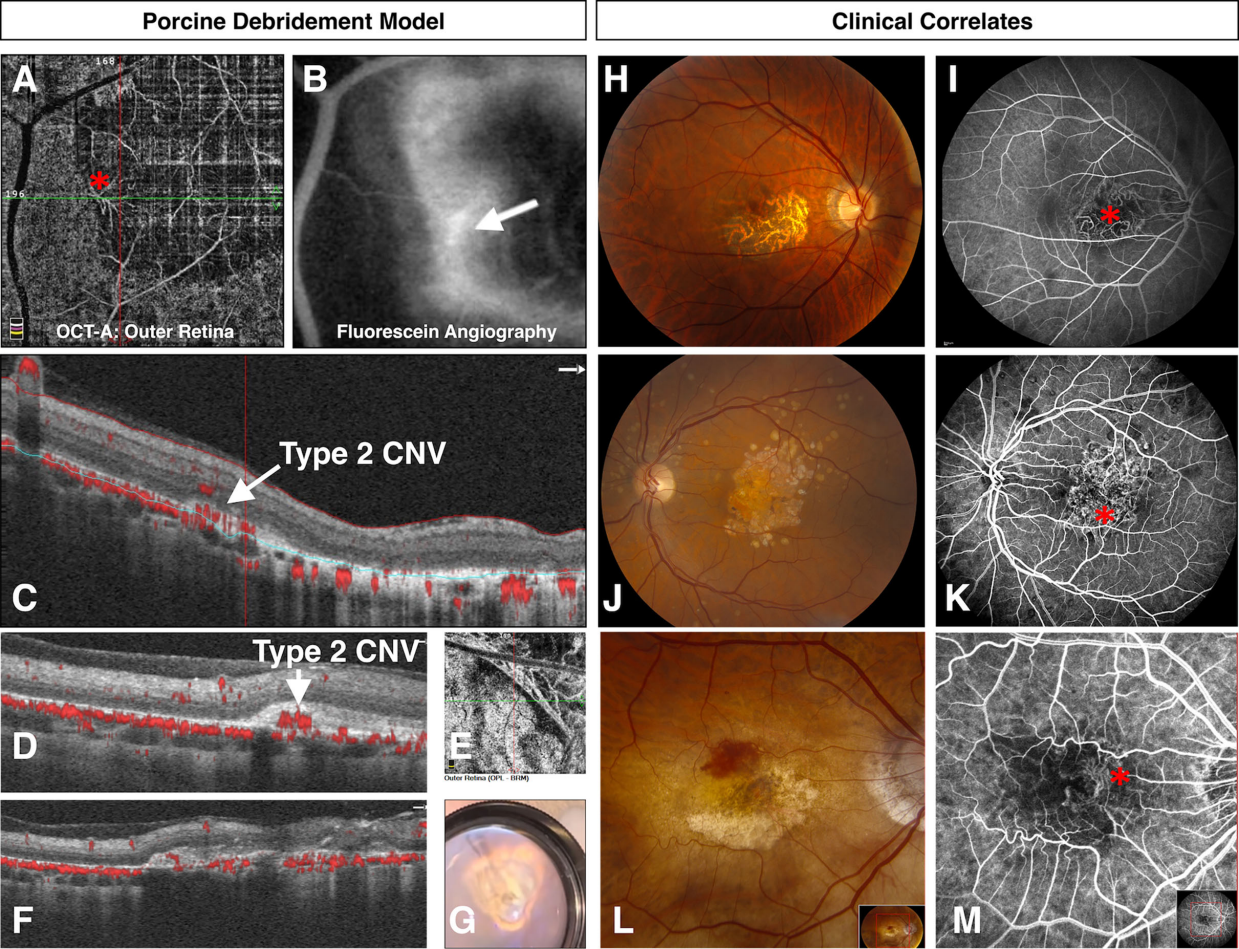
Junctional choroidal neovascular membrane complexes identified in the porcine RPE debridement model with clinical correlations. (**A**–**C**) Two months after debridement surgery, a type 2 (subretinal) choroidal neovascular complex (*red asterisk*) was identified using OCTA (**A**, outer retinal flow), fundus fluorescein angiography (**B**), and structural OCT with OCTA flow overlay (**C**). The CNV complex had increased hyperfluorescence consistent with mild late leakage (white arrow in **B**) on fluorescein angiography testing, confirming exudative process associated with the edge of the atrophic lesion. (**C**) The structural OCT confirmed CNV presence (*white arrow*) at the junctional location between debridement zone and nondebrided region. Note the absence of choriocapillaris flow and underlying pachyvessels present underlying this neovascular complex. **D–G**. A second pig with evidence of type 2 CNV present on OCT/OCTA testing (**E**, **F**) in area of disciform scar, which is noted with indirect ophthalmoscopy (**G**) and OCT testing (**D**, **F**). (**H**–**M**) Imaging from three patients with CNV complexes associated with large GA central areas are shown, including color fundus photography (**H**, **J**, **L**) and fluorescein angiography testing (**I**, **K**, **M**). Patient 1 (**H** and **I**) is a 64-year-old man with PROM1-associated macular dystrophy and filamentous nonexudative CNV (*red asterisk*). Patient 2 (**J** and **K**) is a 62-year-old man with GA, colloidal drusen, and filamentous nonexudative CNV (*red asterisk*). Patient 3 (**L** and **M**) is an 88-year-old woman with GA and exudative CNV on edge of GA (*red asterisk*). Note the hemorrhage seen clinically (**L**).

**Figure 7.**
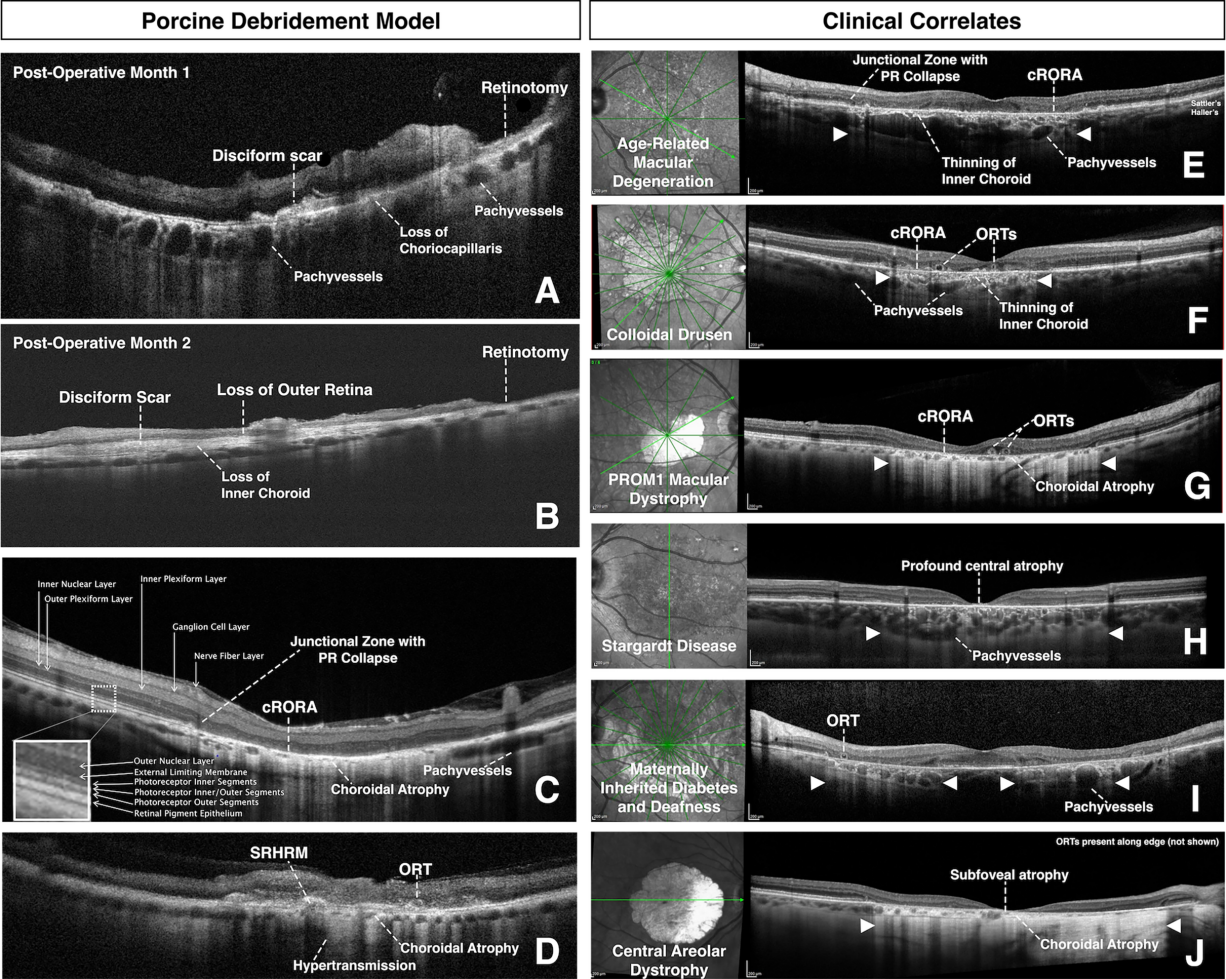
OCT findings identified in the porcine RPE debridement model correlate to several macular diseases. (**A**–**D**) OCT B-scan cross-sections from three different pigs are shown 1 month (**A**) or 2 months (**B**–**D**) postoperatively. (**E**–**J**) OCT B-scan cross-sections with corresponding infrared en face images from six different patients are shown. All patients have different macular diseases, including advanced AMD (**E**), GA-associated with colloidal drusen (**F**), *PROM1*-associated macular dystrophy (**G**), Stargardt disease associated with two pathogenic *ABCA4* variants (**H**), MIDD associated with a pathogenic *MT-TL1* variant (**I**), and central areolar dystrophy associated with a pathogenic *PRPH2* variant (**J**). Specific OCT findings are labeled for all pigs and patients. Note the common features throughout the areas of debridement or GA (*white arrowheads*), including ORTs, cRORA, areas of choroidal atrophy, SRHRM, PR collapse at junctional zone, hypertransmission defects, and pachyvessels. cRORA, complete RPE and outer retina atrophy; MIDD, maternally inherited diabetes and deafness; OCT, optical coherence tomography; ORT, outer retinal tubulation; SRHRM, subretinal hyper-reflective material.

B-scan cross-sectional OCT images and OCTA images were obtained through the debridement zone and outside the debridement zone after surgical debridement of all pigs at week 2 (Fig. 5), month 1, and month 2 (Figs. 2–^3^,4, 5C, 5D, 6, 7) postoperatively. OCT images outside the debridement zone showed normal retinal layers, and corresponding OCTA flow overlay demonstrated normal flow signal throughout the choriocapillaris through nondebrided areas (Figs. 3D, 4B, 5, 6C, 6D, 6F, 7A–7D).

In two pigs (29%), there was a GA phenotype by week 2 with loss of the inner choroidal vasculature in the debridement zone. In these pigs, there was preservation of both inner and outer retinal layers at 2 weeks, (Fig 5B), but by month 2, OCT findings through the debridement area included complete RPE and outer retinal atrophy with associated hypertransmission defects (i.e., light penetration into the choroid) but mostly intact and preserved inner retina (Figs. 2C, 5D, 7C). Figure 5D demonstrates the loss of outer retinal layers that had previously seemed to be preserved at 2 weeks. All pigs (*n* = 7) developed pachyvessels in Sattler's and/or Haller's layers of the choroid, especially in areas near the GA edge (Figs. 2C, 2F, 7A, 7C), and all had evidence of inner choroid thinning on OCT testing (Figs. 3H, 4B, 5B, 5D, 6C–6E, 7A–7D); OCTA flow overlay showed significant choriocapillaris flow loss (*n* = 7) throughout the debridement zone two months after surgery (Figs. 3H, 4B, 5D, 6C). Pigs of both phenotypes (*n* = 4) had complete atrophy of all choroidal layers two months after debridement (Figs. 5D, 6C, 7C, 7D). Partial or complete choroidal atrophy was noted in 100% of debrided eyes shortly after RPE loss and was progressive; the averaged ratio of flow signal area to total area was markedly decreased in the debridement zone (*P* < 0.0001) (Supplementary Fig. S1).

OCT findings in five pigs (71%) included subretinal disciform scar with overlying outer retinal atrophy (Figs. 7A, 7B, 7D); outer retinal tubulations and subretinal hyper-reflective material were also identified in these pigs (Fig. 7D). Figures 7A and 7B show the retinotomy site with distant disciform scar; pachyvessels and choriocapillaris loss beneath the disciform scars in Figures 2F and 7A. Four pigs (57%) developed choroidal neovascularization (CNV) lesions (Figs. 6A–6G), but there was no sign of significant exudation (i.e., no subretinal or intraretinal fluid) noted at any time point for any pig.

### Histological Evaluation of the RPE Debridement Zone

H&E staining identified normal retinal anatomy outside the debridement zone with normal cell density and thickness associated with inner and outer retinal layers (Figs. 3A–3C, 4C). In contrast, histological analysis through the debridement zone was consistent with GA, including significant retinal anatomy changes (Figs. 3E–3G, 4D). In a pig with the disciform scar phenotype, the debrided region at two months postoperatively had evidence of RPE and PR loss, outer retinal disorganization with inner nuclear layer and outer nuclear layer collapse, subretinal deposits, and pigment migration (Figs. 3E–G). Outer retinal tubulation was observed in the area of debridement (Fig. 3F), whereas subretinal material was observed in area of outer retinal loss (Fig. 3G). Comparing Figures 3A and 3E, the choroidal vessels in Haller's layer were larger in the debrided region (i.e., pachyvessels) compared with the nondebrided region. A similar comparison highlights the marked reduction in retinal thickness two months after debridement (Fig. 3). The corresponding OCTA images in Figure 3 corroborate the H&E data showing thinning and loss of vessels of the inner choroid in the debridement zone.

Normal retinal anatomy was observed in pigs with the GA phenotype outside the debridement zone and significant outer retina and choroidal disruption within the debridement zone 2 months after surgery (Fig. 4). Severe choriocapillaris flow loss was observed underneath the debridement zone. Degenerating PRs and collapsing inner nuclear layer and outer nuclear layers in the atrophic region were shared findings between the two phenotypes (Figs. 3G, 4D). The debridement region in all cases exhibited complete loss of RPE and inner choroid, the latter verified with OCTA testing (Fig. 4B).

Immunohistochemistry staining demonstrated that there was Collagen IV staining present at the level of Bruch's membrane both outside and within the area of debridement (Supplementary Figs. S2A–S2B). Residual Bruch's membrane was identified in the debridement zone across all animals tested, even 2 months after debridement in animals noted to have an inflammatory and/or gliosis reaction. Staining with anti-Iba1 showed recruitment of Iba1 positive cells to all layers of the retina and to the subretinal space where the RPE had been removed (Supplementary Figs. S2C–S2D). Similarly, in areas of the junctional zone where there was PR collapse and outer retinal tubulation, there were more glial fibrillary acidic protein–positive cells in the area of debridement compared with the area outside the debridement zone (Supplementary Figs. S2E–S2F).

### CNV Complexes Form in the Junctional Zone After RPE Debridement

Four pigs were noted to develop an MNC, and each had mild late leakage on fluorescein angiography testing in the area of CNV two months after debridement (Fig. 6B). OCTA confirmed the presence of type 2 CNVs (*n* = 4) with increased flow signals in the sub-RPE or subretinal space, respectively. Each CNV complex was noted to form near the junctional zone, where the debridement zone and normal retina transitioned. One pig with GA-associated MNC was followed out to 6 months; this neovascular complex was consistent with a type 2 CNV (Fig. 6).

## Discussion

Of the seven pig eyes described in this study, all developed common features in the areas of RPE debridement that mimicked several age-related and inherited macular diseases (Fig. 7). First, all pigs developed large choroidal vessels in Sattler's and/or Haller's layers, a finding commonly observed in advanced macular degeneration. Ng et al.^29^ found that more than one-half of the eyes (52.1%) with exudative maculopathy owing to AMD without a polypoidal phenotype had findings of pachychoroid, including increased choroidal thickness, choroidal vessels density, and/or vessel diameter (i.e., pachyvessels). These findings suggest that there are structural and functional alterations of the choroid (e.g., choroidal venous stasis, choroidal hyperpermeability) both in the human and the pig model as a direct result of RPE dysfunction. The presence of these vessels, especially in age-related GA, is especially intriguing because histological studies have shown that choroidal vessel size and density decrease with physiological aging.^29^^,^^30^ Overall, the GA-associated pachyvessels and choriocapillaris thinning in this porcine model are consistent with histopathologic findings described in advanced AMD, polypoidal choroidal vasculopathy, and pachychoroid spectrum disorders.^29^^,^^31^

Another common pig OCT finding that simulated human disease included presence of ORTs, which are common in areas of GA, CNV, and/or subretinal hyper-reflective material.^32^ Hypertransmission defects in areas of cRORA, subretinal hyper-reflective material, pigment migration, and disciform scar formation were also found within the debrided regions and were nearly indistinguishable from the human examples of macular degeneration. The collapsed outer retinal layers noted in several pigs, most prominently seen in Figure 4 at the transition zone between GA and healthy retina, seem to be consistent with the external limiting membrane descent described by Li et al.^33^ Histological staining supports the hypothesis that reactive gliosis and disciform scar formations may be due to microglia and astrocyte recruitment to the area of RPE loss.

Before this study, other debridement animal models have been described using various species and both mechanical and chemical debridement techniques. These models have advanced our understanding of GA in the context of macular degeneration and have been effectively used to optimize subretinal cell transplantation. However, models such as that described by Monés et al.^20^ using sodium iodate are not specific to RPE cells. The use of mitomycin C and edetic acid for chemically induced GA was described in the pig in 1995, before the use of OCTA.^18^^,^^19^ Although that study clearly demonstrated with OCT and immunocytochemistry that RPE loss led to choriocapillaris atrophy, RPE repopulation in the debridement zone was a consistent confounding factor and a phenomenon we hoped to mitigate with mechanical debridement. Although both mechanical and laser based methods of debridement have been applied to nonhuman primates and pigs, these studies sought to characterize RPE transplantation immediately following debridement and did not perform a comprehensive analysis of the debridement region absent transplanted RPE.^21^^,^^23^ Our study is the first to do so.

It is important to note that our model shows that RPE loss affects choroidal flow, because a complete flow deficit was noted as early as 2 weeks after RPE debridement. None of the seven pigs had significant subretinal hemorrhage at the time of debridement, suggesting that the consistent and progressive loss of choroid was not due to surgical trauma. One would expect massive bleeding, especially in a pig, if there was significant trauma to the choroid at the time of RPE removal. This finding corroborates clinical OCTA evidence of severe impairment of choriocapillaris flow within the bounds of GA.^34^ However, it is traditionally taught that choroidal circulation changes lead to overlying RPE dysfunction, and eventually death, in advanced AMD and other macular diseases.^5^^,^^29^ Our study shows that the opposite can also occur, where loss of RPE negatively impacts the choroid. These data support the findings of McLeod, et al.^35^ In that study, post mortem analysis of eyes with advanced AMD showed a 50% decrease in choroidal vascular area and capillary constriction in regions of complete RPE atrophy.^35^ Their group concluded that the primary insult in GA is at the level of the RPE, which is consistent with our findings. RPE cells are known to secrete competing pro and antiangiogenic factors (e.g., pigment epithelium-derived factor VEGF. Thus, one hypothesis for RPE loss leading to choroidal atrophy is the potential imbalance between proangiogenic and antiangiogenic factors in the debridement zone.

This porcine model also shows that the choroid atrophies faster than PRs, suggesting that PR loss is, at least partly, owing to choroidal atrophy (i.e., ischemia) and not solely a lack of outer segment phagocytosis. In the work of Li et al.,^33^ histological analysis of GA confirmed end-stage loss of PRs and surrounding supporting structures and vertically oriented cells (e.g., Müller cells). Their study highlights the irreversible tissue damage and loss after RPE loss. Given that our model preserves outer retinal structures initially, it is feasible that immediate replacement of RPE cell sheets could potentially restore structure and function in the debridement area in this pig model. Our laboratories have ongoing translational studies to examine the role of RPE and other cell replacement in this porcine debridement model.

Retinal damage, especially the loss of RPE, has been associated with increased injection pressure during subretinal gene therapy delivery in various animal models, including pigs.^24^ Unfortunately, significant perifoveal chorioretinal atrophy after subretinal gene therapy injection is now recognized as a common complication in human surgeries of voretigeneneparvovec-rzyl (Luxturna), a U.S. Food and Drug Administration–approved gene therapy for patients with biallelic RPE65 variants.^36^^,^^37^ This chorioretinal atrophy in human gene therapy surgeries is not well-understood. Hypotheses include the shearing of RPE owing to increased injection pressures (i.e., significant flow) during bleb propagation and/or viral solution toxicity to the RPE, both of which are plausible explanations. In these cases, which are associated with paracentral scotomas, the RPE, outer retinal layers, and inner choriocapillaris are all thinned or missing, similar to the porcine model we describe here.

Our data, which showed RPE loss first followed by choroid thinning and then PR loss, corroborates the histological findings of Del Priore et al.^18^^,^^19^ Together these studies suggest that the RPE damage from subretinal bleb propagation (from shearing forces) and/or viral toxicity could lead to secondary inner choroid thinning, subsequent outer retinal loss, and eventually choroidal atrophy. Given the junctional, or transitional, zone between the debridement region and nondebrided regions showed significant pathology, including choroidal thinning, pachyvessels, and CNV formation, it is reasonable to believe the choroid-related changes in the junctional zone caused by RPE injury may be responsible for the progressive nature of the perifoveal chorioretinal atrophic regions in human subretinal gene therapy.

Another unsolved and controversial topic in the field of retina is the potential association between anti-VEGF agents and the progression of GA.^38^ Although anti-VEGF therapies have revolutionized the treatment of neovascular (wet) AMD, concerns have been raised regarding their impact on the development or progression of GA. One study showed that central choroidal thickness decreased significantly after anti-VEGF therapy for neovascular AMD, possibly implicating choroidal ischemia and subsequent overlying RPE dysfunction in GA development and/or progression.^39^ Understanding these potential associations necessitates a deeper understanding of the pathophysiology of GA, and future studies exploring this association using this pig model could elucidate the underlying mechanisms of GA expansion.

Because this RPE debridement model results in CNV formation in both the GA and disciform scar phenotypes, future preclinical anti-VEGF studies could be designed using this pig model to test efficacy against CNV formation, exudation, and expansion. It is possible that the nitinol wire used for debridement led to small breaks in Bruch's membrane without resulting in clinically significant hemorrhage at the time of surgery. These small microperforations could eventually lead to CNV development. However, all observed CNVs developed at the junctional zones, which is often observed in patients with GA-associated CNVs. We hypothesize that the areas devoid of RPE underwent choroidal remodeling with loss of choriocapillaris, localized choroidal ischemia, and subsequent CNV formation. It is possible that the alterations in choriocapillaris perfusion and the absence of an effective blood-retina barrier allow leakage of pro-angiogenic factors from RPE at the edges of the debridement zone to the choroid and promote blood vessel growth from the healthy choroid at its edges. Regardless of the etiology, the presence of CNV in this model was both unexpected and welcomed by our team for future research endeavors.

## Supplementary Material

Supplement 1

Supplement 2

Supplement 3
