# Supplementary material for: Early Choriocapillaris Loss in a Porcine Model of RPE Cell Debridement Precedes Pathology That Simulates Advanced Macular Degeneration
Source: Invest Ophthalmol Vis Sci. 2024 Apr 3;65(4):8. doi: 10.1167/iovs.65.4.8 (PMC10996981; doi:10.1167/iovs.65.4.8)
Supplement: Supplement 1 [file iovs-65-4-8_s001.pdf]

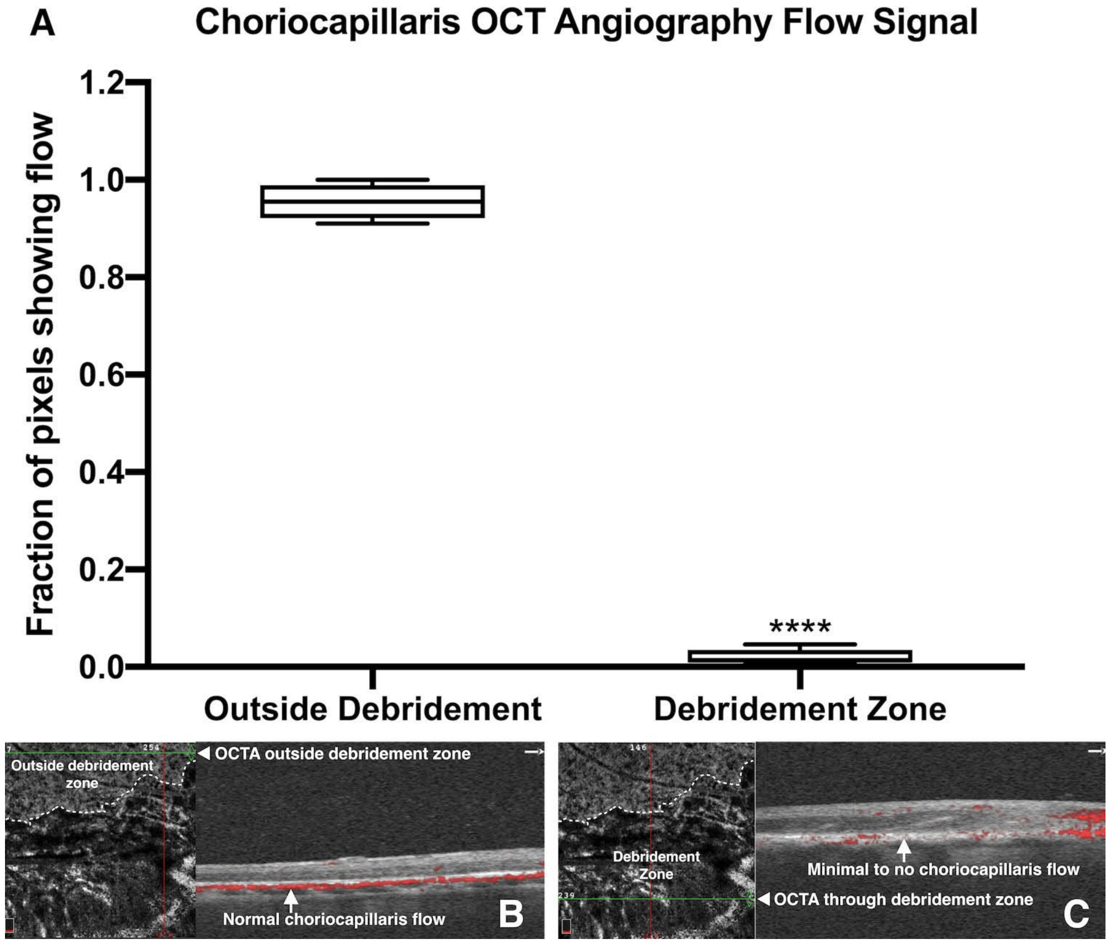

Supplementary Figure 1. Choriocapillaris flow is significantly decreased in debridement zone. **A.** For six pigs, the total area of pixels signaling non-zero flow within a pre-defined 0.5mm diameter (0.2 mm^2) measurement circle was recorded using Optovue flow software within the debridement zone and immediately adjacent to the debridement zone two months after surgery. The averaged ratio of flow signal area to total area was markedly reduced in the debridement zone ($p<0.0001$). **B.** OCT-A choriocapillaris flow show no abnormalities in non-debrided region (green line). Corresponding structural OCT and OCT-A flow overlay demonstrate normal flow signal throughout the choriocapillaris (inner choroid, arrow) through area of non-debrided retina. **C.** OCT-A choriocapillaris flow deficit is noted in area of GA (green line). Corresponding structural OCT and OCT-A flow overlay show significant choriocapillaris flow loss (arrow) throughout debridement zone. GA, geographic atrophy; OCT, optical coherence tomography; OCT-A, OCT-angiography.
