# Supplementary material for: Early Choriocapillaris Loss in a Porcine Model of RPE Cell Debridement Precedes Pathology That Simulates Advanced Macular Degeneration
Source: Invest Ophthalmol Vis Sci. 2024 Apr 3;65(4):8. doi: 10.1167/iovs.65.4.8 (PMC10996981; doi:10.1167/iovs.65.4.8)
Supplement: Supplement 2 [file iovs-65-4-8_s002.pdf]

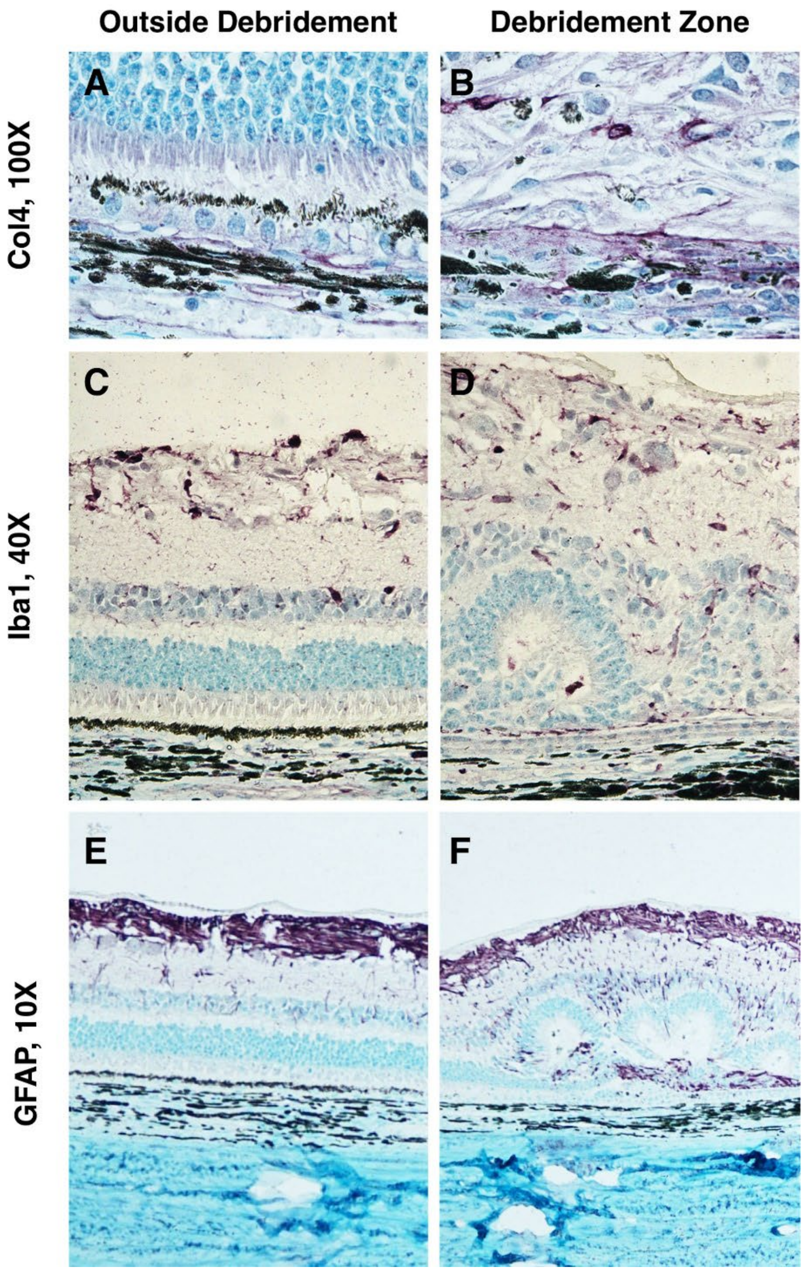


**Supplementary Figure 2. Histological staining demonstrates intact Bruch's membrane and inflammatory cell recruitment in debridement zone**. Col4 staining demonstrates staining outside the debridement zone (**A**) and within the debridement zone (**B**) at the level of Bruch's membrane. For the representative image shown, there was an increase in cell recruitment to the subretinal space with disorganized outer retinal layers in area of RPE loss. Compared to areas outside the debridement zone (**C**), Iba1 staining showed increased Iba1 positive cells in all layers of the retina in the debridement zone (**D**). GFAP positive cells were also identified in all layers of the retina in the debridement zone (**F**) compared to areas outside the debridement zone (**E**). All antibodies were detected using a Vectastain Elite ABC kit with Vector VIP substrate, which generates a purple product. Nuclei were counterstained with Methyl Green.

Col4, collagen type IV; GFAP, glial fibrillary acidic protein; Iba1, ionized calcium binding adaptor molecule 1; RPE, retinal pigment epithelium.
